# Insomnia symptoms and risk of bloodstream infections: prospective data from the prospective population‐based Nord‐Trøndelag Health Study (HUNT), Norway

**DOI:** 10.1111/jsr.13696

**Published:** 2022-09-06

**Authors:** Marianne S. Thorkildsen, Lars E. Laugsand, Tom I. L. Nilsen, Randi M. Mohus, Lise H. Høvik, Tormod Rogne, Erik Solligård, Jan K. Damås, Lise T. Gustad

**Affiliations:** ^1^ Gemini Center for Sepsis Research at Institute of Circulation and Medical Imaging Norwegian University of Science and Technology (NTNU) Trondheim Norway; ^2^ Clinic of Emergency and Prehospital Care St. Olavs hospital Trondheim Norway; ^3^ Department of Circulation and Medical Imaging NTNU Trondheim Norway; ^4^ Clinic of Anaesthesia and Intensive Care St. Olavs Hospital Trondheim Norway; ^5^ Department of Public Health and Nursing NTNU Trondheim Norway; ^6^ Department of Chronic Disease Epidemiology Yale University School of Public Health New Haven Connecticut USA; ^7^ Centre for Fertility and Health Norwegian Institute of Public Health Oslo Norway; ^8^ Centre of Molecular Inflammation Research NTNU Trondheim Norway; ^9^ Department of Clinical and Molecular Medicine NTNU Trondheim Norway; ^10^ Department of Infectious Diseases St. Olavs Hospital Trondheim Norway; ^11^ Faculty of Nursing and Health Sciences Nord University Levanger Norway; ^12^ Department of Medicine and Rehabilitation, Levanger Hospital Nord‐Trøndelag Hospital Trust Levanger Norway

**Keywords:** infection, sepsis, shift work, sleep, sleep and maintenance disorder, work performance

## Abstract

Previous research suggests decreased immune function and increased risk of infections in individuals with insomnia. We examined the effect of insomnia symptoms on risk of bloodstream infections (BSIs) and BSI‐related mortality in a population‐based prospective study. A total of 53,536 participants in the second Norwegian Nord‐Trøndelag Health Study (HUNT2) (1995–97) were linked to prospective data on clinically relevant BSIs until 2011. In Cox regression, we estimated hazard ratios (HRs) with 95% confidence intervals (CIs) for a first‐time BSI and for BSI‐related mortality (BSI registered ≤30 days prior to death) associated with insomnia symptoms. Compared with participants who reported “no symptoms”, participants reporting having “difficulty initiating sleep” (DIS) often/almost every night had a HR for a first‐time BSI of 1.14 (95% CI 0.96–1.34). Participants reporting “difficulties maintaining sleep” (DMS) often/almost every night had a HR of 1.19 (95% CI 1.01–1.40), whereas those having a feeling of “non‐restorative sleep” once a week or more had a HR of 1.23 (95% CI 1.04–1.46). Participants frequently experiencing all three of the above symptoms had a HR of 1.39 (1.04–1.87), whilst those who had both DIS and DMS had a HR of 1.15 (0.93–1.41) and being troubled by insomnia symptoms to a degree that affected work performance was associated with a HR of 1.41 (95% CI 1.08–1.84). The HRs for BSI‐related mortality suggest an increased risk with increasing insomnia symptoms, but the CIs are wide and inconclusive. We found that frequent insomnia symptoms and insomnia symptoms that affected work performance were associated with a weak positive increased risk of BSI.

## INTRODUCTION

Insomnia is a sleep disorder defined as having a subjective feeling of difficulties initiating or maintaining sleep, or early morning awakening and impaired daytime functioning. Insomnia affects from 6% to 19% of the European population (Riemann et al., 2017), and women have a higher predisposition for insomnia than men (Zhang & Wing, 2006). Sleep is a restorative process important for the immune system and insomnia can be detrimental to both the innate and adaptive immune system (Besedovsky et al., 2019) and affect genes for immune functioning (Irwin, 2019). Biological mechanisms related to the effect of insomnia on immune responses include elevated C‐reactive protein levels (Meier‐Ewert et al., 2004), decreased natural killer cell activity (Irwin et al., 1994), suppressed interleukin 2 (IL‐2) and IL‐6 response (Burgos et al., 2006; Vgontzas et al., 2009), decreased T‐cell cytokine production (Irwin et al., 1996), reduced vaccination response (Lange et al., 2003), and impaired performance of the pro‐inflammatory cytokine genes IL‐6 mRNA and tumour necrosis factor alpha (TNFα) mRNA (Irwin et al., 2006), reduced vaccination response (Lange et al., 2003) and impaired performance of the pro‐inflammatory cytokine genes IL‐6 mRNA and TNFα mRNA (Irwin et al., 2006).

Previous studies investigating the association of sleep with risk of infections have mainly focused on the risk of respiratory infections (Cohen et al., 2009; Lin et al., 2018; Patel et al., 2012; Prather et al., 2015; Prather & Leung, 2016) and report that participants with sleep disorders and short sleep duration were more susceptible to respiratory infections especially in the younger adult age groups (Mackenzie, 2016). Whether insomnia is associated with the risk of other and more severe invasive infections, such as bloodstream infections (BSIs) is not clear. BSI is a serious infectious disease, rating among the top seven causes of deaths in Europe and North America (Goto & Al‐Hasan, 2013), men are more at risk (Randi Marie Mohus et al., 2022), and BSI often leads to sepsis (Laupland et al., 2005). Sepsis is defined as a dysregulated host response to infection, estimated to annually afflict 48.9 million people and cause 11 million deaths worldwide (Rudd et al., 2020). Insomnia is thought to affect the risk of all‐cause mortality and cardiovascular disease mortality (Pienaar et al., 2021); however, we are not aware of studies assessing if insomnia symptoms are associated with BSI risk and BSI‐related mortality.

In this study, we prospectively examined if three insomnia symptoms mirroring *Diagnostic and Statistical Manual of Mental Disorders*, fourth edition (DSM‐IV) and two insomnia symptoms mirroring DSM‐IV criteria was associated with the risk of clinically relevant BSI with verified microbes (Mehl et al., 2017), as well as with risk of BSI‐related mortality using data from the population‐based Nord‐Trøndelag Health Study (HUNT) in Norway. The microbiology data allowed us to also examine the association of insomnia symptoms and risk of specific bacterial BSIs, such as *Staphylococcus* (*S*.) *aureus*, *Escherichia* (*E*.) *coli* and *Streptococcus* (*S*.) *pneumonia*.

## METHODS

### Study population

**FIGURE 1:**
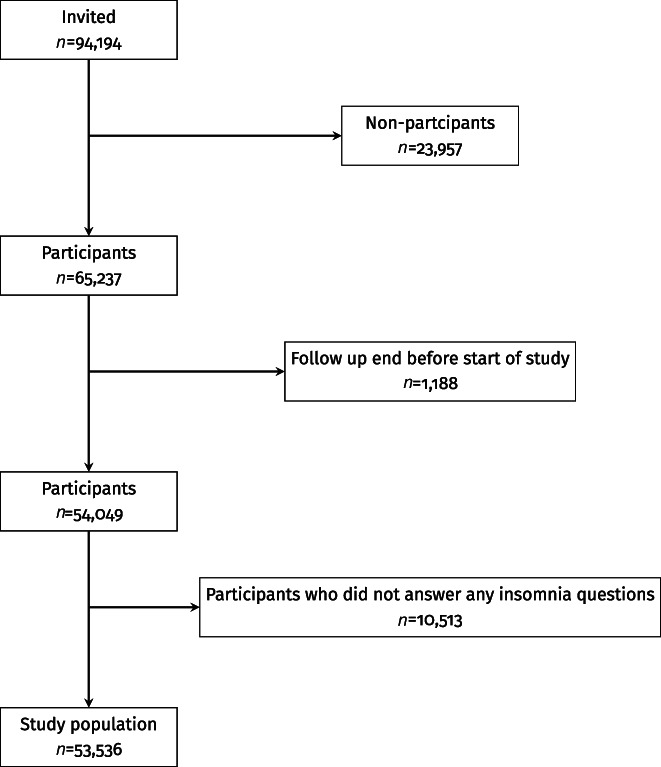
Flow chart of participant inclusion

This study is based on data from the second HUNT study in Norway (HUNT2) conducted in 1995–1997. All inhabitants aged ≥20 years (*n* = 93,989) in the geographical region of Nord‐Trøndelag were invited to participate, and 65,237 (69.5%) attended the survey, completed extensive questionnaires and attended for a clinical examination (Krokstad et al., 2013). Of these, 1,188 participants were excluded due to either having a first BSI event, death, or migration before start of follow‐up (for details see outcome ascertainment). Moreover, 10,513 (16.1%) participants did not answer any of the insomnia symptom questions, leaving a total of 53,536 individuals in the analytical sample (Figure 1).

### Outcome ascertainment

The HUNT2 data were linked to the hospital‐based sepsis registry in Nord‐Trøndelag Hospital Trust (HNT HF) by the unique 11‐digit Norwegian identification number. The HNT HF sepsis registry prospectively collects information on all BSIs that require hospitalisation at the two community hospitals, Levanger Hospital (from 1994) and Namsos Hospital (from September 1999) or at the tertiary referral hospital St. Olavs hospital, Trondheim (from 1994). We defined our outcome variables as first‐time BSI, and first‐time BSI by the most common bacteria; *E. coli*, *S. aureus* and *S. pneumoniae*. Our definition of BSI‐related mortality was a BSI registered ≤30 days prior to death, as this is previously established as the “gold standard” outcome for deaths from BSIs requiring hospital stays (Laupland et al., 2019). For individuals with multiple positive blood cultures, a new episode of BSI was defined as a positive blood culture >30 days after the previous episode. Isolates only consisting of Coagulase negative *Staphylococcus species*, *Corynebacterium species* and *Cutibacterium species* were not considered as BSI, as these bacteria are associated with contamination of blood cultures with bacterial skin flora (Paulsen et al., 2017). The participants were followed‐up until they experienced an event or until censoring by death, emigration, or end of follow‐up December 31, 2011.

### Insomnia

The HUNT2 insomnia symptom questionnaire mirrored the DSM‐IV criteria and included three items, of which two items were reflected in the DSM‐V. One question was related to “difficulty initiating sleep” (DIS; “Have you had difficulties falling asleep in the last month”) and another related to “difficulties maintaining sleep” (DMS; “During the last month, have you woken up too early and not been able to get back to sleep”). Both questions had the response options “never” (0 points), “occasionally” (1 point), “often” (2 points), and “almost every night” (3 points) and still remains in the DSM‐V criteria for insomnia. The third question was related to “non‐restorative sleep” (NRS; “How often do you suffer from poor sleep, i.e., non‐restorative sleep”), with the response option “never or few times a year” (0 points), “one to two times per month” (1 point), “about once a week” (2 points), “more than once a week” (3 points). NRS was removed from the DSM‐V in order to distinguish more clearly between insomnia and other sleep disorders, e.g., obstructive sleep apnea, where NRS is a common symptom (Chung et al., 2015; Olufsen et al., 2020). This item is also often the most present item (39.9%) in individuals with insomnia versus DIS present in 14% and DMS in 32% (Chung et al., 2015). For the present study, we thus kept this item in order to be able to compare with previous research. For each item, 0 points were defined as the reference (no insomnia symptoms), 1 point as the middle value, and 2 or 3 points were merged into the highest category (frequent insomnia symptoms). All participants aged ≥20 years (*n* = 53,536) were asked about DIS and DMS, whereas NRS was only determined in participants aged 20–69 years (*n* = 44,271). We summarised into two cumulative insomnia symptom scores, one based on the sum‐score on DIS, DMS, NRS (mirroring DSM‐IV insomnia) and one based on the sum‐score on DIS, DMS (mirroring DSM‐V insomnia). Each item gave 0 points to the cumulative insomnia score if no symptoms where present, or else it gave 1 point. Cumulative insomnia thus ranged from zero to three insomnia items present for the DSM‐IV‐like items and zero to two for the DSM‐V‐like items. Calculation of the cumulative insomnia score related to the DSM‐IV‐like items was only performed among those without any missing data on each insomnia item, as only participants aged <70 years were asked if they had a feeling of NRS.

Participants aged 20–69 years (*n* = 44,271) were also asked if symptoms related to sleep influenced their work performance (“During the last year, have you been troubled by insomnia to such a degree that it influenced your work performance”, with the response option “yes” or “no”).

### Covariates

#### Demographic factors

Age and sex were obtained from the National Population Registry at the time of HUNT2 participation. Other sociodemographic factors were self‐reported. We categorised marital status into “never married”, “married”, or “separated/widower/divorced” (Hauan et al., 2018). Education level was categorised as “<10 years”, “10–12 years” and “>12 years” of fulfilled schooling (Askim et al., 2018). Participants answering “yes” to being employed, self‐employed or working full‐time in the home was categorised as “working”. If they answered “yes” to being retired or unemployed, they were categorised as “not working”. Participants answered “yes” or “no” to whether they worked shift, night or on call.

#### Clinical information

Weight (kg) and height (cm) was measured by trained nurses while the participants wore light clothes and no shoes and rounded to the closest half kilogrammes and centimetres (Holmen et al., 2003). Body mass index (BMI) was then calculated from weight in kilogrammes (kg) divided by the squared value of height (m^2^) and categorised according to World Health Organization (WHO) recommendations; <18.5, 18.5–24.9, 25–29.9, 30.0–34.9, 35.0–39.9 and ≥40.0 kg/m^2^ (Mohus et al., 2018).

#### Lifestyle factors

Weekly alcohol consumption was based on participants’ report of how many units of beer, wine, and liquor they usually consumed in a 2‐week period. The amount of beer, wine and liquor units was combined and categorised as “abstainer” (0 units per week or answering “yes” to being an abstainer), “light drinker” (1–14 units), “moderate drinker” (15–28 units) and “heavy drinker” (>28 units) (Paulsen et al., 2017). A total of 10% of the participants did not answer the questions related to alcohol intake. Some of these participants did answer how many days a month they on average consumed alcohol, and this information was added to reduce missing data. Answering “zero times a month” was included in the “abstainer” group, “one–three times a month” in “light drinker”, “four–seven times a month” as “moderate drinker” and “more than seven times a month” as “heavy drinker”.

Smoking habits was categorised as “never”, “former”, or “current smoker”. In addition, physical activity was categorised into four groups based on how many hours of light and hard activity they performed. Light activity was defined as activity not involving sweating or feeling of breathlessness. Participants performing neither hard nor light activity were categorised as “inactive”, <3 h of light activity and no hard activity as “light activity”, ≥3 h of light or <3 h of hard activity as “moderate activity”, and >3 h of hard activity as “vigorous activity” (Paulsen et al., 2017).

#### Chronic somatic disorders

Self‐reported information regarding comorbid chronic disorders was summarised into one variable answering yes/no to having at least one condition. Cardiovascular disease (CVD) was defined as having a history of acute myocardial infarction, angina pectoris, stroke, or surgery for intermittent claudication. Participants answering “yes” to having had productive cough continuously for >3 months every year for the last 2 years were defined as having lung disease. Cancer and diabetes diagnosis were defined by participants answering “yes” to having/or having had a diagnosis. Rheumatoid disease was defined as answering “yes” to having ankylosis spondylitis and/or rheumatoid arthritis. Participants with missing information on each of the comorbid conditions were categorised as not having the specific comorbid conditions.

#### Sleeping medication and circadian factors

As the HUNT survey is close to the Arctic Circle, being examined in the months April through August was categorised as “high light exposure”, from October through February as “low light exposure”, and March and September as “neither high nor low light exposure” (Sivertsen et al., 2009). We defined chronic use of sedatives as using sedative one or more times a week.

#### Depression and anxiety symptoms

Depression and anxiety symptom levels were self‐reported and assessed using the Hospital Anxiety and Depression Scale (HADS) (Zigmond & Snaith, 1983). The scale consists of 14 items, seven questions related to depression symptoms and seven related to anxiety symptoms. The scale on each item ranges from 0 to 21, with increasing scores indicating increasing symptom load. Per protocol, we replaced one–two missing items on the HADS depression and HADS anxiety subscale with six/seven and five/seven of the values provided (Askim et al., 2018) and defined these as our complete case.

#### Laboratory measurements

Non‐fasting serum blood samples was drawn from each HUNT participant by trained study nurses and analysed at the Central Laboratory at Levanger Hospital. Creatinine levels were measured using the Jaffe method with sample blank (Roche Diagnostics, Mannheim, Germany). Chronic kidney disease was defined as having an estimated glomerular filtration rate (eGFR) of <60 ml/min per 1.73 m^2^. The eGFR was estimated using the Modification of Diet in Renal Disease (MDRD)‐formula, from re‐calibrated creatinine values (Hallan et al., 2006).

### Statistical analysis

We imputed missing data using multiple imputation by fully conditional specification generating a total of 10 complete datasets in the “mi chained”‐routine and compared these with results from complete case. Information on 12 variables from demography, social status, health behaviours, mental health, and time to event were used as predictor variables to ensure the required assumption of missing at random. Cox regression was used to estimate the hazard ratio (HR) with 95% confidence interval (CI) for first‐time BSI, first‐time BSI stratified by specific bacteria, and BSI‐related mortality associated with each of the insomnia symptoms and cumulative insomnia symptoms. For all comparisons, participants with no insomnia complaints were used as the reference group. All estimated effects were adjusted for first age (as the time scale), which constitutes the crude analysis. Thereafter, we added adjustments for sex, marital status, education, physical activity, alcohol consumption, BMI, and smoking habits. Factors that could be mediators of the association between insomnia and risk of BSI, such as common chronic disorders, depression or anxiety symptoms were adjusted for in sensitivity analyses. Among the 44,271 participants in employment, we evaluated whether sleep problems influencing work performance was associated with risk of BSI after additional adjustment for shift work. Possible linear trend across insomnia categories were assessed in analysis where the insomnia categories (0–3) for the sum score of DIS/DMS/NRS categories and (0–2) for the sum‐score of DIS/DMS were entered as continuous variables in the regression model. The proportional hazards assumptions were evaluated by Schoenfeld residuals and visual inspection of log–log plots. Censoring information on death or migration was obtained from the National Population Register. As risk of infection and BSI varies by age and sex, possible effect modification by age (±60 years) and sex (man, woman) was evaluated in a likelihood ratio test of a product term of insomnia and age, and of insomnia and sex.

We performed several sensitivity analyses to address the robustness of our findings. To reduce possible influence by reverse causation we excluded the first 5 years of follow‐up. We evaluated whether varying light exposure from seasonal changes influenced our results by stratifying the population by circadian light exposure on time of insomnia assessment in HUNT2. We stratified those who reported insomnia symptoms by months with daylight predominance. Lastly, the potential effect of use of sedatives was examined by assessing the risk of first‐time BSI only among those who reported no use of sedatives.

The statistical analyses were performed using STATA, version 16 (Stata Corp., College Station, TX, USA ).

### Ethics

Participation in the HUNT2 survey is voluntary, and participants are always able to withdraw from the health survey by contacting the HUNT research centre. All information will then be deleted, and blood and urine samples annihilated. All participants signed a written consent to data collection and to linkage of data to other registers. This study was approved by the Regional Ethics Committee for Medical Research (REK 2018/1819/REKmidt and REK 2012/153), and by the HUNT Data Access Committee. From the Data Access Committee in HNT HF, we had approval to use and merge the HUNT data file with the HNT sepsis registry.

## RESULTS

During a median follow‐up of 14.9 years, 1,579 (3.0%) of the participants had an episode of BSI and 284 (0.5%) BSI‐related mortality. Baseline characteristics of the participants are shown in Table 1.

**TABLE 1:** Baseline characteristics of the study population at inclusion in the second Norwegian Nord‐Trøndelag Health Study (HUNT2), according to difficulties initiating sleep

	Missing, *n*	Difficulties initiating sleep		
		Never	Occasionally	Often/almost every night
*N* (%):				
Sex (female)	0	14,601 (50.3)	11,128 (58.0)	3,101 (66.7)
Smoking (current)	975	7,046 (24.7)	5,667 (30.1)	1,762 (38.8)
Marital status (married)	114	18,163 (62.6)	11,651 (60.8)	2,690 (58.0)
Education (10–12 years)	2,370	12,616 (45.2)	7,785 (42.5)	1,592 (36.8)
Alcohol consumption (light)	1,618	13,906 (49.2)	8,639 (46.5)	1,729 (38.8)
Physical activity (light)	5,159	7,729 (29.2)	5,619 (32.5)	1,468 (37.0)
Working shifts (yes)	16,342	4,255 (20.2)	2,842 (22.1)	577 (22.2)
Comorbidity		6,526 (80,9)	1,189 (13.2)	460 (5.1)
CVD	32	1,812 (6.2)	1,602 (8.4)	653 (14.1)
CKD	113	1,008 (3.5)	863 (4.5)	310 (6.7)
Lung disease	282	2,653 (9.2)	2,096 (11.0)	736 (16.0)
Cancer	2,651	897 (3.2)	769 (4.3)	272 (6.5)
Diabetes	98	757 (2.6)	576 (3.0)	212 (4.6)
Rheumatic disease	4,875	916 (3.4)	874 (5.1)	325 (8.5)
Use of sleep medicine weekly	5,270	590 (2.3)	1,448 (8.3)	1,721 (40.6)
Mean (SD):				
Age, years	0	48.4 (16.7)	51.5 (16.9)	55.5 (17.3)
BMI, kg/m^2^	291	26.3 (4.0)	26.4 (4.0)	26.6 (4.4)
Depression score (0–21)	1,484	2.8 (2.7)	3.9 (3.0)	5.6 (3.8)
Anxiety score (0–21)	2,260	3.4 (2.8)	4.9 (3.3)	7.0 (4.3)

BMI, body mass index; CVD, cardiovascular disease; CKD, chronic kidney disease; SD, standard deviation.

The HR for first‐time BSI associated with each of the insomnia items and cumulative insomnia symptom score are presented in Table 2. Compared with participants reporting “no symptoms of insomnia”, participants experiencing DIS “often/almost every night” had a HR of 1.14 (95% CI 0.96–1.34); and DMS had a HR of 1.19 (95% CI 1.01–1.40) for BSI. Participants with NRS once a week or more had a HR of 1.23 (95% CI 1.04–1.46). Having all three cumulative symptoms reflected in the DSM‐IV insomnia definition was associated with a HR of 1.39 (95% CI 1.04–1.87), while having both DIS and DMS was associated with a HR of 1.15 (95% CI 0.93–1.41) for BSI compared to having none of the insomnia symptoms. Moreover, the employed participants who responded “yes” to “being troubled by insomnia to such a degree that it affected work performance” had a HR of 1.41 (95% CI 1.08–1.84) for BSI, compared to the employed whom responded “no” to the question (Table 3).

**TABLE 2:** Risk of a first‐time bloodstream infection event associated with insomnia symptoms

Symptom	Person years	BSI events, *n*	HR^a^	HR^b^	95% CI
DIS					
Never	375,494	759	1.00	1.00	Reference
Occasionally	243,288	599	1.00	1.06	0.95–1.18
Often/almost every night	55,458	182	1.08	1.14	0.96–1.34
Linear trend^c^	674,239	1540	1.03	1.06	0.99–1.15
DMS					
Never	328,670	491	1.00	1.00	Reference
Occasionally	285,548	837	1.03	1.10	0.98–1.23
Often/almost every night	61,320	230	1.10	1.19	1.01–1.40
Linear trend^c^	675,537	1,558	1.04	1.09	1.01–1.18
NRS^d^					
Never, few times a year	408,694	534	1.00	1.00	Reference
1–2 times/month	98,093	166	1.10	1.15	0.96–1.37
Once a week/more than once a week	88,069	198	1.21	1.23	1.04–1.46
Linear trend^c^	594,855	898	1.10	1.11	1.03–1.21
Cumulative symptoms (DIS, DMS, NRS)					
0	479,449	640	1.00	1.00	Reference
1	57,854	117	1.12	1.13	0.93–1.38
2	35,241	75	1.20	1.20	0.94–1.53
3	16,512	50	1.40	1.39	1.04–1.87
Linear trend ^d^	589,055	882	1.11	1.11	1.03–1.20
Cumulative symptoms (DIS, DMS)					
0	579,654	1,227	1.00	1.00	Reference
1	65,938	195	1.04	1.06	0.91–1.24
2	24,470	101	1.10	1.15	0.93–1.41
Linear trend^c^	670,062	1,523	1.05	1.07	0.98–1.17

BMI, body mass index (kg/m^2^); CI, confidence interval; DIS, difficulty initiating sleep; DMS, difficulty maintaining sleep; HR, hazard ratio; NRS, non‐restorative sleep.

^a^ Age‐adjusted: adjusted for age (as the timescale).

^b^ Multivariably adjusted: adjusted for age (as the timescale), sex (male, female), marital status (married/partner, separated/divorced/widower or never married), education (<10, 10–12, >12 years), BMI (<18.5, 18.5–24.9, 25–29.9, 30.0–34.9, 35.0–39.9 and ≥40.0 kg/m^2^), smoking (never, former, current), alcohol consumption (abstainer, light drinker, moderate drinker, heavy drinker), physical activity (inactive, light activity, moderate activity, vigorous activity).

^c^ Estimates when entering the insomnia categories (0–3) for DIS/DMS/NRS and (0–2) for DIS/DMS as a continuous variable in the regression model.

^d^ Analysis restricted to age group 20–69 years (*n* = 44,671).

**TABLE 3:** Risk of first‐time bloodstream infection event related to insomnia affecting work performance^a^

Work performance^a^ ^,^ ^d^							
Symptom	Person years	Events, *n*	HR^b^	HR^c^	95% CI	HR^c^	95% CI
No	410,632	410	1.00	1.00	Reference	1.00	Reference
Yes	45,837	63	1.41	1.41	1.08–1.84	1.41	1.08–1.83

CI, confidence interval; HR, hazard ratio.

^a^ Work performance was measured in participants aged 20–69 years (*n* = 44,271) by the question: “During the last year, have you been troubled by insomnia to such a degree that it influenced your work performance”, with the response option (“yes” or “no”).

^b^ Age‐adjusted: adjusted for age (as the timescale).

^c^ Multivariably adjusted: adjusted for age (as the timescale), sex (male, female), marital status (married/partner, separated/divorced/widower or never married), education (<10, 10–12, >12 years), body mass index(<18.5, 18.5–24.9, 25–29.9, 30.0–34.9, 35.0–39.9 and ≥40.0 kg/m^2^), smoking (never, former, current), alcohol consumption (abstainer, light drinker, moderate drinker, heavy drinker), physical activity (inactive, light activity, moderate activity, vigorous activity).

^d^ Participants were asked whether they “during the last year, have you been troubled by insomnia to such a degree that it affected your work?”. Analysed in the population that worked and answered the question in the questionnaire (*n* = 33,044).

In sensitivity analyses, adjustment for shift work and doing our analysis in complete case had no effect on the results (data not shown). When adjusting for potentially mediating factors (comorbid conditions and HADS anxiety and depression scores) of the associations between insomnia symptoms and risk of BSI the HRs were somewhat attenuated (Table S1).

Table S2 shows the risk of BSI by specific bacteria. Regarding *E. coli* BSI, in analysis of linear trend, the HRs were 1.18 (95% CI 1.04–1.34) for DSM and 1.12 (95% CI 1.00–1.27) for DIS. This was not observed in analysis for linear trend for DIS/DMS/NRS or DIS/DMS cumulative insomnia symptoms or for any insomnia symptoms related to *S. pneumonia or S. aureus*. For *S. pneumonia*, the HR pointed towards increased risk when having difficulties maintaining sleep and having a feeling of non‐restorative sleep often or almost every night, but CIs were also in line with no difference.

Of the total 1,579 participants experiencing BSI, 283 (18.0%) suffered BSI‐related mortality. Table 4 shows the HRs of BSI‐related mortality according to categories of insomnia symptoms. The risk estimates for BSI‐related mortality associated with insomnia symptoms were similar to the estimates observed for first‐time BSI. For example, compared to the reference category, the HRs for those having the three cumulative insomnia symptoms DIS/DMS/NRS was 1.45 (95% CI 0.72–2.93). However, due to relatively few BSI‐related deaths CIs were wide and inconclusive.

**TABLE 4:** Risk of a bloodstream infection‐related death associated with insomnia symptoms

Symptom	Person years	Events, *n*	HR^a^	HR^b^	95% CI
DIS					
Never	375,494	138	1.00	1.00	Reference
Occasionally	243,288	109	0.96	1.04	0.80–1.34
Often/almost every night	55,458	26	0.77	0.85	0.55–1.31
Linear trend^c^	674,239	273	0.90	0.96	0.80–1.15
DMS					
Never	328,670	82	1.00	1.00	Reference
Occasionally	285,548	152	0.94	1.06	0.81–1.40
Often/almost every night	61,320	47	1.08	1.29	0.89–1.86
Linear trend^c^	675,537	281	1.03	1.12	0.94–1.35
NRS^d^					
Never, few times a year	408,694	75	1.00	1.00	Reference
1–2 times/month	98,093	21	0.95	0.99	0.61–1.62
Once a week/more than once a week	88,069	35	1.37	1.40	0.92–2.12
Linear trend^c^	594,855	131	1.15	1.17	0.95–1.44
Cumulative symptoms (DIS, DMS, NRS)^d^					
0	479,449	91	1.00	1.00	Reference
1	57,854	21	1.28	1.30	0.80–2.10
2	35,241	9	0.94	0.93	0.47–1.86
3	16,512	9	1.55	1.45	0.72–2.93
Linear trend^c^	589,055	130	1.11	1.09	0.90–1.33
Cumulative symptoms (DIS, DMS)					
0	579,654	218	1.00	1.00	Reference
1	65,938	34	0.95	1.01	0.70–1.45
2	24,470	18	0.97	1.08	0.66–1.77
Linear trend^c^	670,061	270	0.98	1.03	0.83–1.28

BMI, body mass index (kg/m^2^); CI, confidence interval; DIS, difficulty initiating sleep; DMS, difficulty maintaining sleep; HR, hazard ratio; NRS, non‐restorative sleep.

^a^ Age‐adjusted: adjusted for age (as the timescale).

^b^ Multivariably adjusted: adjusted for age (as the timescale), sex (male, female), marital status (married/partner, separated/divorced/widower or never married), education (<10, 10–12, >12 years), BMI (<18.5, 18.5–24.9, 25–29.9, 30.0–34.9, 35.0–39.9 and ≥40.0 kg/m^2^), smoking (never, former, current), alcohol consumption (abstainer, light drinker, moderate drinker, heavy drinker), physical activity (inactive, light activity, moderate activity, vigorous activity).

^c^ Estimates when entering the insomnia categories (0–3) for DIS/DMS/NRS and (0–2) for DIS/DMS as a continuous variable in the regression model.

^d^ Analysis restricted to age group 20–69 years (*n* = 44,671).

In the sensitivity analysis examining the effect of light exposure at the time of reported insomnia symptoms on BSI outcomes, we observed no appreciable changes in our results (data not shown). When we analysed risk of BSI in the population not using sedatives on a weekly basis, the risk was higher for participants with three cumulative insomnia symptoms of DIS/DMS/NRS than for the total population, with a 56% (95% CI 6%–131%) increased risk (Table S3). We observed no notable differences in risk estimates when investigating the influence of reverse causation (Table S4).

When assessing effect modification by age (±60 years) and sex, we found no evidence of interaction in any of the analysis (all *p* > 0.2).

## DISCUSSION

In this prospective population‐based study of 53,536 individuals, we observed weak positive associations between all measures of insomnia symptoms mirroring the DSM‐IV and DSM‐V criteria and risk of BSI. People who reported three cumulative insomnia symptoms of DIS, DSM and NRS had 40% increased risk of future BSI compared to participants having no insomnia symptoms. Among the working population, those who reported that their insomnia affected their ability to work were also at 40% increased risk of BSI. The results for BSI‐related mortality were inconclusive.

A previous retrospective study following 24,173 Taiwanese participants for 10 years, found 143% increased risk of pneumonia among participants with international statistical classification of diseases and related health problems (ICD)‐9 diagnosis of insomnia, compared with participants without this diagnosis (Lin et al., 2018). As the ICD‐9 and DSM‐IV are mirroring similar insomnia problems, we do not think our access to the DSM‐IV, instead of the DSM‐V could explain the difference. As Lin et al. investigated pneumonia and we investigated BSI, the higher effect estimates could be due to different outcomes and adjustments for less confounders than in our study.

Results from the bacteria‐specific analyses point in the direction of insomnia symptoms being an increased risk for future BSI of *E. coli*. These results regarding *E. coli* coincide with previous studies having found sleep‐deprived rabbits to be more likely to get a positive blood culture, have higher morbidity and mortality when exposed to *E. coli*, than rabbits with enhanced sleep (Toth et al., 1993). However, as Toth et al. (1993) found the risk for BSI in rabbits also related to *S. aureus*, it seems most plausible that our negative results for other bacteria are due to few events and thus lower precision.

The risk estimates for BSI‐related mortality with the insomnia items pointed towards a 40% increased risk but with imprecise estimates due to few events. However, a meta‐analysis that found short sleep duration resulted in a higher all‐cause mortality can give support to the plausibility of our effect estimates (Gallicchio & Kalesan, 2009), especially as short sleep duration might be similar to our items regarding difficulty initiating sleep or difficulty maintain sleep.

The similar BSI risk with seasonal variations in light exposure could be explained by insomnia being a stable trait at 63–65° north where the HUNT study is performed and also further north near the polar circle (Shochat et al., 2019; Sivertsen et al., 2020). Interpreting the 50% increased risk with having all three DSM‐IV symptoms in the population not using sedatives versus the 40% increased risk in the total population is difficult as we do not know whether the participants answered how their sleep is with or without sedatives. Our study should not be taken into account of a reduced risk of BSI with sleeping medication without further investigations, as previous studies have shown that some sleep medications increase risk of infection (Huang et al., 2014; Kripke, 2016).

In the studies most comparable to ours, they either lacked information regarding comorbid conditions (Cohen et al., 2009; Prather et al., 2015), were limited to a female population or they were not able to adjust for other potential important confounders (Lin et al., 2018; Prather & Leung, 2016). In contrast our study was not sex restricted and included both presumably healthy and comorbid participants, and we were also able to adjust for important lifestyle factors. Previous research on preventable risk factors of BSI has found: a HR of 1.78 with a BMI of 30 kg/m^2^ compared to normal weight (Rogne et al., 2020); smokers had a HR of 1.51 compared to non‐smokers, and inactive individuals had a 71% HR compared to active individuals; (Paulsen et al., 2017); men had a 41% higher risk for first‐time BSI compared to women (Mohus et al., 2022); BMI and lifestyle (Askim et al., 2018); a HR of 1.72 with low serum iron (Mohus et al., 2018) compared to normal levels, a HR of 1.09 for thyroid‐stimulating <0.5 mU/L compared to 0.5–1.4 mU/L (Thorkildsen et al., 2022). Individuals with severe depression symptoms had a HR of 1.38 and 1.48 for severe anxiety symptoms, all partially explained by comorbidities (Askim et al., 2018). In our study the relative risks were slightly attenuated after adjustment for HADS, but we cannot disentangle if this is due to confounding or mediation effects of mental health problems. We tested for reverse causality by excluding the first 5 years of follow‐up and found no notable changes in risk estimates.

### Strength and limitations

Major strengths of the present study include the large population with a prospective design and minimal loss to follow‐up. Another strength is the use of a confirmed severe invasive infection by blood culture as our outcome as opposed to self‐reported infections (Patel et al., 2012; Prather & Leung, 2016). As an example, in pneumonia, the phenotype of the infectious agent is often unknown, and the heterogeneity of the disease is known to be large. Due to the rich baseline information in HUNT2, we were able to adjust analyses for important lifestyle factors (smoking, alcohol consumption, BMI, and activity level) that were not available in previous studies (Lin et al., 2018). However, as with any observational study limitations include the possibility for residual confounding due to poorly measured, unmeasured, or unknown factors influencing both exposure and disease. Moreover, there is also a potential for including mediators of the association and thus underestimating the true effect of the exposure (Rothman et al., 2008). Based on subject matter knowledge, the most likely confounders are sex and age, while lifestyle factors, comorbidities, anxiety and depression symptoms have more unclear causal pathways. Insomnia could both be a result of or be worsened by poor somatic health, and it could worsen both mental and physical health (Besedovsky et al., 2019; Grandner, 2020). These factors where therefore not included in the main analysis, but rather adjusted for in supplementary sensitivity analyses to assess the robustness of our results.

Other limitations include the lack of DSM‐IV or ‐V‐specific diagnostic criteria for insomnia, as the HUNT insomnia symptom questionnaire did not include the item “early morning awakening” from the DSM‐V and only asked for symptoms the last month and not for longer durations. However, it is known that prevalence of insomnia is lower in studies using the DSM‐IV compared to studies with less strict definitions. As Edinger & Means (2005) point out, the use of a strict criteria for prevalence may cause an underestimation of clinically important sleep problems. We also do not have information on whether the participants’ sleeping pattern is a temporary situation or their regular habit, as insomnia was only evaluated at the start of the study. Lastly, no evaluation of sleep apnea syndrome was available.

## CONCLUSION

This is the first human‐based study to date regarding insomnia as a risk factor for future BSI and BSI‐related mortality. We found evidence of a weak positive increased risk of first‐time BSI with all the DSM‐IV‐ and two of the DSM‐V‐related insomnia symptoms as well as moderately increased risk with cumulative insomnia symptoms and insomnia to such a degree that it affected work performance. More research is needed to understand if the same applies when having access to all the DSM‐V insomnia criteria and to understand the underlying mechanisms causing increased risk of BSI with increasing symptoms of insomnia. As BSI is a global concern, addressing modifiable risk factors such as insomnia is of utmost importance to reduce the burden of BSI. By understanding this causal relationship, we could possibly prevent BSI in the future by addressing insomnia as a preventable risk factor.

## AUTHOR CONTRIBUTIONS

Marianne S. Thorkildsen, Lars E. Laugsand, Tom I. L. Nilsen and Lise T. Gustad made substantial contributions to conception and design; Marianne S. Thorkildsen, Randi M. Mohus and Lise T. Gustad contributed to acquisition of data; all authors contributed to analysis and interpretation of data; Marianne S. Thorkildsen and Lise T. Gustad drafted the manuscript; all authors revised it for critical content and approved the final version of the manuscript.

## FUNDING INFORMATION

Our work was supported by the Faculty of Medicine at the NTNU.

## CONFLICTS OF INTEREST

None of the authors have any conflicts of interest to declare.

## Supporting information


**Table S1** Risk of first‐time BSI event, adjusted for comorbidities and HADS depression and anxiety score, associated with insomnia symptoms
**Table S2**. Risk of first‐time event BSI event specified by bacteria.
**Table S3.** Risk of first‐time BSI event in a population reportion to not use sedatives on a regular basis
**Table S4.** Risk of first‐time BSI event adjusted for comorbidities excluding the first 5 years of follow‐up

## Data Availability

Data available on request due to privacy/ethical restrictions
