# Supplementary material for: Insomnia symptoms and risk of bloodstream infections: prospective data from the prospective population‐based Nord‐Trøndelag Health Study (HUNT), Norway
Source: J Sleep Res. 2022 Sep 6;32(1):e13696. doi: 10.1111/jsr.13696 (PMC10078600; doi:10.1111/jsr.13696)
Supplement: Supplementary file 1 — Table S1 Risk of first‐time BSI event, adjusted for comorbidities and HADS depression and anxiety score, associated with insomnia symptoms Table S2. Risk of first‐time event BSI event specified by bacteria. Table S3. Risk of first‐time BSI event in a population reportion to not use sedatives on a regular basis Table S4. Risk of first‐time BSI event adjusted for comorbidities excluding the first 5 years of follow‐up [file JSR-32-0-s001.docx]

| **Supplementary Table 1.** Risk of first-time BSI event, adjusted for comorbidities and HADS depression and anxiety score, associated with insomnia symptoms. | | | | | | | | | | |
| --- | --- | --- | --- | --- | --- | --- | --- | --- | --- | --- |
| **Symptom** | **Person-years** | **Events** | **HR^a^** | **95% CI** | **HR^b^** | **95% CI** | **HR^c^** | **95% CI** | **HR^d^** | **95% CI** |
| **DIS** |  |  |  |  |  |  |  |  |  |  |
| Never | 375,494 | 759 | 1.00 | Reference | 1.00 | Reference | 1.00 | Reference | 1.00 | Reference |
| Occasionally | 243,288 | 599 | 1.00 | 0.90-1.11 | 1.06 | 0.95-1.18 | 1.03 | 0.93-1.15 | 1.02 | 0.91-1.14 |
| Often/almost every night | 55,458 | 182 | 1.08 | 0.92-1.27 | 1.14 | 0.96-1.34 | 1.07 | 0.90-1.26 | 1.04 | 0.87-1.24 |
| Linear trend^f^ | 674,239 | 1,540 | 1.03 | 0.95-1.11 | 1.06 | 0.99-1.15 | 1.03 | 0.96-1.11 | 1.02 | 0.94-1.11 |
| **DMS** |  |  |  |  |  |  |  |  |  |  |
| Never | 328,670 | 491 | 1.00 | Reference | 1.00 | Reference | 1.00 | Reference | 1.00 | Reference |
| Occasionally | 285,548 | 837 | 1.03 | 0.92-1.15 | 1.10 | 0.98-1.23 | 1.08 | 0.96-1.21 | 1.07 | 0.95-1.21 |
| Often/almost every night | 61,320 | 230 | 1.10 | 0.94-1.29 | 1.19 | 1.01-1.40 | 1.12 | 0.95-1.32 | 1.10 | 0.93-1.31 |
| Linear trend^f^ | 675,537 | 1,558 | 1.04 | 0.97 | 1.09 | 1.01-1.18 | 1.06 | 0.98-1.15 | 1.05 | 0.97-1.14 |
| **NRS^e^** |  |  |  |  |  |  |  |  |  |  |
| Never, few times a year | 408,694 | 534 | 1.00 | Reference | 1.00 | Reference | 1.00 | Reference | 1.00 | Reference |
| 1-2 Times per month | 98,093 | 166 | 1.10 | 0.93-1.31 | 1.15 | 0.96-1.37 | 1.13 | 0.95-1.34 | 1.11 | 0.93-1.33 |
| Once a week/ more than once a week | 88,069 | 198 | 1.21 | 1.02-1.42 | 1.23 | 1.04-1.46 | 1.17 | 0.99-1.39 | 1.15 | 0.96-1.37 |
| Linear trend^f^ | 594,855 | 898 | 1.10 | 1.01-1.19 | 1.11 | 1.03-1.21 | 1.09 | 1.00-1.18 | 1.07 | 0.98-1.17 |
| **Cumulative symptoms (DIS, DMS, NRS)^e^** |  |  |  |  |  |  |  |  |  |  |
| 0 | 479,449 | 640 | 1.00 | Reference | 1.00 | Reference | 1.00 | Reference | 1.00 | Reference |
| 1 | 57,854 | 117 | 1.12 | 0.92-1.36 | 1.13 | 0.93-1.38 | 1.10 | 0.90-1.34 | 1.08 | 0.88-1.32 |
| 2 | 35,241 | 75 | 1.20 | 0.95-1.53 | 1.20 | 0.94-1.53 | 1.15 | 0.90-1.46 | 1.11 | 0.86-1.42 |
| 3 | 16,512 | 50 | 1.40 | 1.05-1.87 | 1.39 | 1.04-1.87 | 1.30 | 0.97-1.74 | 1.23 | 0.91-1.68 |
| Linear trend^f^ | 589,055 | 882 | 1.11 | 1.03-1.20 | 1.11 | 1.03-1.20 | 1.08 | 1.00-1.17 | 1.07 | 0.98-1.16 |
| **Cumulative symptoms (DIS, DMS)**^e^ |  |  |  |  |  |  |  |  |  |  |
| 0 | 579,654 | 1,227 | 1.00 | Reference | 1.00 | Reference | 1.00 | Reference | 1.00 | Reference |
| 1 | 65,938 | 195 | 1.04 | 0.89-1.21 | 1.06 | 0.91-1.24 | 1.02 | 0.88-1.19 | 1.01 | 0.86-1.18 |
| 2 | 24,470 | 101 | 1.11 | 0.90-1.36 | 1.15 | 0.93-1.41 | 1.07 | 0.87-1.32 | 1.04 | 0.84-1.29 |
| Linear trend^f^ | 670,062 | 1,523 | 1.05 | 0.96-1.14 | 1.07 | 0.98-1.17 | 1.03 | 0.94-1.13 | 1.02 | 0.92-1.12 |

Abbreviations: DIS=Difficulty initiating sleep, DMS=Difficulty maintaining sleep, NRS=Non restorative sleep, HR=Hazard ratio, CI=Confidence interval, BSI=Bloodstream infection, BMI=Body mass index (kg/m^2^).

^a^ Age-adjusted: Adjusted for age (as the timescale).

^b^ Multivariably adjusted: (as the timescale) and sex (male, female), marital status (married/partner, separated/divorced/widower or never married), education (<10, 10–12, >12 years), BMI (< 18.5, 18.5– 24.9, 25–29.9, 30.0–34.9, 35.0–39.9 and ≥40.0 kg/m2), smoking (never, former, current), alcohol consumption (abstainer, light drinker, moderate drinker, heavy drinker), physical activity (inactive, light activity, moderate activity, vigorous activity).

^c^ Multivariable + comorbidities adjusted: Multivariable adjusted and adjusted for comorbid conditions (chronic vascular disease, chronic kidney disease, lung disease, cancer, diabetes and rheumatic disease).

^d^ Multivariable + comorbidities and HADS score adjusted: Multivariable and comorbidities adjusted and adjusted for HADS anxiety and depression score.

^e^ Analysis performed in the age group 20 to 69 (n=44,671).^f^ Estimates when entering the insomnia categories (0–3) for DIS/DMS/NRS and (0–2) for DIS/DMS as a continuous variable in the regression model.

| **Supplementary Table 2**. Risk of first-time event BSI event specified by bacteria. | | | | | | | | | | |
| --- | --- | --- | --- | --- | --- | --- | --- | --- | --- | --- |
|  |  | **E. Coli** | | | **S. Pneumonia** | | | **S. Aureus** | | |
| **Symptom** | **Person-years** | **Events** | **HR^a^** | **95% CI** |  | **HR^a^** | **95% CI** |  | **HR^a^** | **95% CI** |
| **DIS** |  |  |  |  |  |  |  |  |  |  |
| Never | 375,494 | 257 | 1.00 | Reference | 105 | 1.00 | Reference | 97 | 1.00 | Reference |
| Occasionally | 243,288 | 242 | 1.18 | 0.99-1.41 | 74 | 0.95 | 0.71-1.29 | 63 | 0.91 | 0.66-1.25 |
| Often/almost every night | 55,458 | 74 | 1.22 | 0.93-1.59 | 20 | 0.93 | 0.57-1.51 | 20 | 1.01 | 0.62-1.66 |
| Linear trend^c^ | 674,239 | 573 | 1.12 | 1.00-1.27 | 199 | 0.96 | 0.77-1.19 | 180 | 0.97 | 0.78-1.21 |
| **DMS** |  |  |  |  |  |  |  |  |  |  |
| Never | 328,670 | 150 | 1.00 | Reference | 69 | 1.00 | Reference | 62 | 1.00 | Reference |
| Occasionally | 285,548 | 345 | 1.36 | 1.01-1.65 | 107 | 1.13 | 0.82-1.55 | 96 | 1.02 | 0.73-1.41 |
| Often/almost every night | 61,320 | 90 | 1.33 | 1.02-1.74 | 26 | 1.12 | 0.70-1.78 | 22 | 0.92 | 0.56-1.52 |
| Linear trend^c^ | 675,537 | 585 | 1.18 | 1.04-1.34 | 202 | 1.08 | 0.86-1.34 | 180 | 0.97 | 0.77-1.23 |
| **NRS^b^** |  |  |  |  |  |  |  |  |  |  |
| Never, few times a year | 408,694 | 179 | 1.00 | Reference | 77 | 1.00 | Reference | 65 | 1.00 | Reference |
| 1-2 Times per month | 98,093 | 61 | 1.16 | 0.86-1.55 | 25 | 1.23 | 0.78-1.94 | 13 | 0.78 | 0.43-1.43 |
| Once a week/ more than once a week | 88,069 | 76 | 1.21 | 0.92-1.60 | 28 | 1.29 | 0.83-2.02 | 22 | 1.12 | 0.68-1.84 |
| Linear trend^c^ | 594,855 | 316 | 1.11 | 0.97-1.27 | 130 | 1.15 | 0.92-1.42 | 100 | 1.03 | 0.80-1.32 |
| **Cumulative symptoms (DIS, DMS, NRS)^b^** |  |  |  |  |  |  |  |  |  |  |
| 0 | 479,449 | 214 | 1.00 | Reference | 97 | 1.00 | Reference | 75 | 1.00 | Reference |
| 1 | 57,854 | 49 | 1.31 | 0.95-1.79 | 18 | 1.22 | 0.73-2.02 | 10 | 0.83 | 0.43-1.61 |
| 2 | 35,241 | 28 | 1.20 | 0.80-1.79 | 10 | 1.07 | 0.55-2.06 | 6 | 0.80 | 0.35-1.86 |
| 3 | 16,512 | 19 | 1.35 | 0.84-2.18 | 5 | 0.97 | 0.39-2.42 | 7 | 1.57 | 0.71-3.49 |
| Linear trend^c^ | 589,055 | 310 | 1.12 | 0.99-1.27 | 130 | 1.03 | 0.83-1.28 | 98 | 1.05 | 0.82-1.33 |
| **Cumulative symptoms (DIS, DMS)** |  |  |  |  |  |  |  |  |  |  |
| 0 | 579,654 | 452 | 1.00 | Reference | 163 | 1.00 | Reference | 151 | 1.00 | Reference |
| 1 | 65,938 | 75 | 1.04 | 0.82-1.33 | 26 | 1.09 | 0.72-1.66 | 15 | 0.67 | 0.39-1.14 |
| 2 | 24,470 | 40 | 1.09 | 0.78-1.51 | 10 | 0.94 | 0.49-1.79 | 12 | 1.13 | 0.62-2.06 |
| Linear trend^c^ | 670,062 | 567 | 1.04 | 0.90-1.21 | 199 | 1.01 | 0.78-1.32 | 178 | 0.94 | 0.71-1.25 |

Abbreviations: DIS=Difficulty initiating sleep, DMS=Difficulty maintaining sleep, NRS=Non restorative sleep, HR=Hazard ratio, CI=Confidence interval, BSI=Bloodstream infection, BMI=Body mass index (kg/m^2^).

^a^ Multivariably adjusted: adjusted for age (as the timescale), sex (male, female), marital status (married/partner, separated/divorced/widower or never married), education (<10, 10–12, >12 years), BMI (< 18.5, 18.5– 24.9, 25–29.9, 30.0–34.9, 35.0–39.9 and ≥40.0 kg/m2), smoking (never, former, current), alcohol consumption (abstainer, light drinker, moderate drinker, heavy drinker), physical activity (inactive, light activity, moderate activity, vigorous activity).

^b^ Analysis restricted to age group 20 to 69 years (n=44,671).

^c^ Estimates when entering the insomnia categories (0–3) for DIS/DMS/NRS and (0–2) for DIS/DMS as a continuous variable in the regression model.

| **Supplementary Table 3.**  Risk of first-time BSI event in a population reportion to not use sedatives on a regular basis. | | | | | |
| --- | --- | --- | --- | --- | --- |
|  | **Person-years** | **Events** | **HR^a^** | **HR^b^** | **95% CI** |
| **DIS** |  |  |  |  |  |
| Never | 330,409 | 655 | 1.00 | 1.00 | Reference |
| Occasionally | 203,945 | 459 | 0.97 | 1.04 | 0.92-1.17 |
| Often/almost every night | 31,950 | 81 | 1.18 | 1.24 | 0.98-1.56 |
| Linear trend^d^ | 566,304 | 1,195 | 1.03 | 1.08 | 0.98-1.18 |
| **DMS** |  |  |  |  |  |
| Never | 289,799 | 428 | 1.00 | 1.00 | Reference |
| Occasionally | 235,679 | 638 | 0.99 | 1.07 | 0.94-1.21 |
| Often/almost every night | 41,249 | 142 | 1.14 | 1.24 | 1.02-1.51 |
| Linear trend^d^ | 566,727 | 1,208 | 1.05 | 1.10 | 1.01-1.21 |
| **NRS**^c^ |  |  |  |  |  |
| Never, few times a year | 361,521 | 467 | 1.00 | 1.00 | Reference |
| 1-2 Times per month | 84,691 | 136 | 1.07 | 1.16 | 0.96-1.41 |
| Once a week/ more than once a week | 62,987 | 123 | 1.14 | 1.23 | 1.02-1.48 |
| Linear trend^d^ | 509,199 | 726 | 1.07 | 1.10 | 1.00-1.21 |
| **Cumulative symptoms (DIS, DMS, NRS)^c^** |  |  |  |  |  |
| 0 | 423,884 | 558 | 1.00 | 1.00 | Reference |
| 1 | 47,4598 | 88 | 1.06 | 1.09 | 0.87-1.37 |
| 2 | 24,856 | 44 | 1.10 | 1.12 | 0.82-1.53 |
| 3 | 9,215 | 27 | 1.53 | 1.56 | 1.06-2.31 |
| Linear trend^d^ | 505,554 | 717 | 1.10 | 1.11 | 1.01-1.23 |
| **Cumulative symptoms (DIS, DMS)** |  |  |  |  |  |
| 0 | 503,625 | 1,020 | 1.00 | 1.00 | Reference |
| 1 | 47,895 | 117 | 0.99 | 1.01 | 0.83-1.22 |
| 2 | 12,288 | 50 | 1.43 | 1.50 | 1.13-2.00 |
| Linear trend^d^ | 563,808 | 1,187 | 1.11 | 1.14 | 1.01-1.29 |

Abbreviations: DIS=Difficulty initiating sleep, DMS=Difficulty maintaining sleep, NRS=Non restorative sleep, HR=Hazard ratio, CI=Confidence interval, BSI=Bloodstream infection, BMI=Body mass index (kg/m^2^).

^a^ Age-adjusted: Adjusted for age (as the timescale).

^b^ Multivariably adjusted: Age-adjusted (as the timescale) and sex (male, female), marital status (married/partner, separated/divorced/widower or never married), education (<10 years, 10–12 years, >12 years), BMI (< 18.5, 18.5– 24.9, 25–29.9, 30.0–34.9, 35.0–39.9 and ≥40.0 kg/m^2^), smoking (never, former, current), alcohol consumption (abstainer, light drinker, moderate drinker, heavy drinker), physical activity (inactive, light activity, moderate activity, vigorous activity).

^c^ Analysis performed in the age group 20 to 69 (n=43.198).

^d^ Estimates when entering the insomnia categories (0–3) for DIS/DMS/NRS and (0–2) for DIS/DMS as a continuous variable in the regression model.

| **Supplementary Table 4** – Risk of first-time BSI event adjusted for comorbidities excluding the first five years of follow up. | | | | | |
| --- | --- | --- | --- | --- | --- |
| **Symptom** | **Person-years** | **Events** | **HR^a^** | **HR^b^** | **95% CI** |
| **DIS** |  |  |  |  |  |
| Never | 258,912 | 595 | 1.00 | 1.00 | Reference |
| Occasionally | 166,953 | 475 | 1.00 | 1.07 | 0.95-1.21 |
| Often/almost every night | 37,364 | 131 | 1.02 | 1.08 | 0.89-1.31 |
| Linear trend^d^ | 463,229 | 1,201 | 1.01 | 1.05 | 0.96-1.14 |
| **DMS** |  |  |  |  |  |
| Never | 227,416 | 390 | 1.00 | 1.00 | Reference |
| Occasionally | 195,216 | 650 | 1.01 | 1.07 | 0.94-1.22 |
| Often/almost every night | 41,396 | 176 | 1.08 | 1.16 | 0.97-1.39 |
| Linear trend^d^ | 464,027 | 1,216 | 1.03 | 1.08 | 0.98-1.18 |
| **NRS**^c^ |  |  |  |  |  |
| Never, few times a year | 286,385 | 440 | 1.00 | 1.00 | Reference |
| 1-2 Times per month | 68,378 | 138 | 1.11 | 1.16 | 0.96-1.41 |
| Once a week/ more than once a week | 61,435 | 162 | 1.19 | 1.23 | 1.02-1.48 |
| Linear trend^d^ | 416,435 | 740 | 1.09 | 1.11 | 1.02-1.22 |
| **Cumulative symptoms (DIS, DMS, NRS)**^c^ |  |  |  |  |  |
| 0 | 335,735 | 528 | 1.00 | 1.00 | Reference |
| 1 | 40,458 | 98 | 1.13 | 1.15 | 0.92-1.43 |
| 2 | 24,567 | 57 | 1.10 | 1.11 | 0.85-1.47 |
| 3 | 11,498 | 42 | 1.43 | 1.42 | 1.03-1.96 |
| Linear trend^d^ | 412,197 | 725 | 1.10 | 1.10 | 1.01-1.20 |
| **Cumulative symptoms (DIS, DMS)** |  |  |  |  |  |
| 0 | 399,336 | 970 | 1.00 | 1.00 | Reference |
| 1 | 44,841 | 143 | 0.97 | 1.00 | 0.84-1.19 |
| 2 | 16,357 | 76 | 1.09 | 1.14 | 0.90-1.44 |
| Linear trend^d^ | 460,534 | 1,189 | 1.02 | 1.05 | 0.941-1.16 |

Abbreviations: DIS=Difficulty initiating sleep, DMS=Difficulty maintaining sleep, NRS=Non restorative sleep, HR=Hazard ratio, CI=Confidence interval, BSI=Bloodstream infection, BMI=Body mass index (kg/m^2^).

^a^ Age-adjusted: Adjusted for age (as the timescale).

^b^ Multivariably adjusted: Adjusted for age (as the timescale), sex (male, female), marital status (married/partner, separated/divorced/widower or never married), education (<10, 10-12, >12 years), BMI (< 18.5, 18.5– 24.9, 25–29.9, 30.0–34.9, 35.0–39.9 and ≥40.0 kg/m^2^), smoking (never, former, current), alcohol consumption (abstainer, light drinker, moderate drinker, heavy drinker), physical activity (inactive, light activity, moderate activity, vigorous activity).

^c^ Analysis performed in the age group 20 to 69 (n=44,671).

^d^ Estimates when entering the insomnia categories (0–3) for DIS/DMS/NRS and (0–2) for DIS/DMS as a continuous variable in the regression model.
